# Body sway reflects joint emotional expression in music ensemble performance

**DOI:** 10.1038/s41598-018-36358-4

**Published:** 2019-01-18

**Authors:** Andrew Chang, Haley E. Kragness, Steven R. Livingstone, Dan J. Bosnyak, Laurel J. Trainor

**Affiliations:** 1 Department of Psychology, Neuroscience and Behaviour, McMaster University, Hamilton, ON L8S 4K1 Canada; 2 McMaster Institute for Music and the Mind, McMaster University, Hamilton, ON L8S 4K1 Canada; 3 Rotman Research Institute, Baycrest Hospital, Toronto, ON M6A 2E1 Canada

## Abstract

Joint action is essential in daily life, as humans often must coordinate with others to accomplish shared goals. Previous studies have mainly focused on sensorimotor aspects of joint action, with measurements reflecting event-to-event precision of interpersonal sensorimotor coordination (e.g., tapping). However, while emotional factors are often closely tied to joint actions, they are rarely studied, as event-to-event measurements are insufficient to capture higher-order aspects of joint action such as emotional expression. To quantify joint emotional expression, we used motion capture to simultaneously measure the body sway of each musician in a trio (piano, violin, cello) during performances. Excerpts were performed with or without emotional expression. Granger causality was used to analyze body sway movement time series amongst musicians, which reflects information flow. Results showed that the total Granger-coupling of body sway in the ensemble was higher when performing pieces with emotional expression than without. Granger-coupling further correlated with the emotional intensity as rated by both the ensemble members themselves and by musician judges, based on the audio recordings alone. Together, our findings suggest that Granger-coupling of co-actors’ body sways reflects joint emotional expression in a music ensemble, and thus provide a novel approach to studying joint emotional expression.

## Introduction

Joint action is essential to everyday life. Humans regularly coordinate with each other to achieve shared goals, ranging from moving an object too heavy for one person to playing on a sports team. The prevalence of sophisticated joint actions in our daily lives has led to widespread investigation of the psychological, social, and neural mechanisms implicated in the execution of these actions^1–3^.

Previous studies have largely focused on sensorimotor aspects of interpersonal coordination^4–6^, and the cooperation and group affiliation that synchronized movement facilitates^7,8^, even in infants^9,10^. However, it has been proposed that felt emotions and empathy can affect joint actions^6,11^. Even in cognitive cooperation, a study using the joint Simon task demonstrated that participants integrated co-actors’ task representations into their own, but only if they had a positive, cooperative relationship with that co-actor^12^. Neuroscientific studies show that positive emotion facilitates nonverbal vocalizations and activates premotor cortex, part of the network supporting interpersonal interaction^13^. Further, one study found that pianists with higher empathy scores had a better motor representation of a duet co-performer, as reflected by motor-evoked potentials^14^. Together, these studies show that emotion influences sensorimotor joint action. However, to the best of our knowledge, no studies have explicitly investigated joint emotional expression, in which coordinating emotional expression amongst individuals is one of the primary goals of the shared activity.

The performing arts represent one area in which joint emotional expression is essential. Emotional expression is a central goal in music performances^15,16^, and performers often depart from the notated score to communicate emotions and musical structure by introducing microvariations in intensity and speed^17,18^. Music ensemble performers therefore must coordinate not only their actions, but also their joint expressive goals^19^. For musicians in an ensemble, sharing a representation of a global performance outcome facilitates joint music performance^20,21^. Interpersonal event-to-event temporal precision has been widely used as a local index of sensorimotor aspects of joint action^22–24^. However, this method is likely insufficient to capture higher-order aspects of joint performance, which may involve stylistic asynchronies, complex leader-follower dynamics, and expressive variations in timbre, phrasing, and dynamics, which take place over longer time scales and are not necessarily reflected by event-to-event temporal precision. For example, a previous study examined the inter-onset intervals of piano duet keystrokes, but cross-correlation analysis failed to reveal leader-follower relationships, likely because these depend on aspects of joint performance involving longer time scales^25^.

Body sway among co-actors might be a useful measurement of joint emotional expression. Body sway is a domain-general index for measuring real-time, real-world interpersonal coordination and information sharing. Relations between co-actors’ body sway have been associated with joint action performance in many domains, including engaging in motor coordination tasks^26,27^, having a conversation^28–30^, and music ensemble performance^25,31–34^. Specifically in music performance, it has been associated with melodic phrasing^35^, suggesting it reflects the higher-order aspect of music performance, rather than lower-order note-to-note precision.

In a previous study, we experimentally manipulated leadership roles in a string quartet and examined the predictive relationships amongst the performers’ body sway movements^36^. Results showed that leaders’ body sway more strongly predicted other musicians’ body sway than did the body sway of followers, suggesting that body sway coupling reflects directional information flow. This effect was diminished, but still observed, even when musicians could not see each other, suggesting that body sway is, at least in part, a byproduct of psychological processes underlying the planning and production of music. This process is similar to how gestures during talking reflect thoughts and facilitate speech production, in addition to being directly communicative^37^. Furthermore, the total coupling strength in a quartet (averaged amount of total predictive movement across each pair of performers) positively correlated with performers’ self-ratings of performance quality, but it did not necessarily correlate with self-ratings of synchronization. This suggests that body sway coupling might reflect performance factors above and beyond interpersonal temporal precision (synchronization), and might reflect in part emotional expression.

A music ensemble is an ideal model for investigating joint emotional expression^38–40^. First, though emotion may be important in joint action more generally, emotional expression is an essential goal of music performance. In addition, group coordination is a universal feature of music ensembles across human cultures, suggesting that interpersonal interaction is an important component of musical engagement^41^. Finally, ensemble music performance shares many psychological principles with other forms of interpersonal coordination, making the findings generalizable to other domains^42^.

To quantify the magnitude of interpersonal joint emotional expression in a music ensemble, we used both Granger causality and cross-correlation to analyze the body sway coupling among the performers. Granger causality is a statistical estimation of the degree to which one time series is predicted by the history of another time series, over and above prediction by its own history. The larger the value of Granger causality, the better the prediction, and the more information that is flowing from one time series to another. Previous studies have shown that Granger causalities among performers’ motions in a music ensemble reflect leadership dynamics and thus information flow^31,36,43^, which are higher-order aspects of joint action. On the other hand, cross-correlation analysis is a measure of the similarity between two time series as a function of different time delays between the two series^44^ and has been used to examine motion synchrony among co-actors^23,25,45–47^. Cross-correlation analysis also appears to associate with higher-order aspects of interpersonal interactions, as temporal similarity between co-actors’ body motions has been shown to affect group affiliation and to modulate interpersonal social cooperation^48–51^.

These two measures, Granger causality and cross-correlation, appear to reflect different aspects of interpersonal coupling, and it is unclear whether information flow or similarity underlies joint emotional expression. We hypothesized that information flow (represented by Granger causality of body sway coupling, or “Granger-coupling”) would be crucial for joint emotional expression, because it reflects the dynamic interpersonal communication needed for achieving joint musical performance^36^. On the other hand, we hypothesized that similarity (represented by cross-correlation of body sway coupling, or “correlational-coupling”) would not be associated with joint emotional expression. Supporting this idea, our previous study showed that Granger-coupling but not correlational-coupling among performers’ body sway reflected leadership dynamics^36^, suggesting that correlational-coupling might not detect the higher-order outcomes of interpersonal coordination, such as emotional expression and leader-follower relationships.

In the current study, we used a professional piano trio, including a pianist, a violinist, and a cellist, as a model to investigate whether interpersonal joint emotional expression is reflected by body sway coupling between co-actors. The trio performers played six happy and six sad excerpts, each played with and without emotional expression on different trials. The performers’ body sways were recorded with a passive optical motion capture system. After each performance, the musicians were asked to rate their group performance on expressivity and synchronization. We recruited eleven additional professional musician judges to rate the performances based on audio recordings. We hypothesized that (1) the total Granger-coupling of body sway among performers would be higher when performing music pieces with emotional expression as compared to without emotional expression, reflecting greater information flow across the ensemble, and (2) the Granger-coupling of body sway would positively correlate with the rated degree of emotional expression. Additionally, we examined whether correlation-coupling of body sway also reflected joint emotional expression.

## Results

### Granger-coupling of body sway, but not correlational-coupling, reflects joint emotional expression

**Figure 1:**
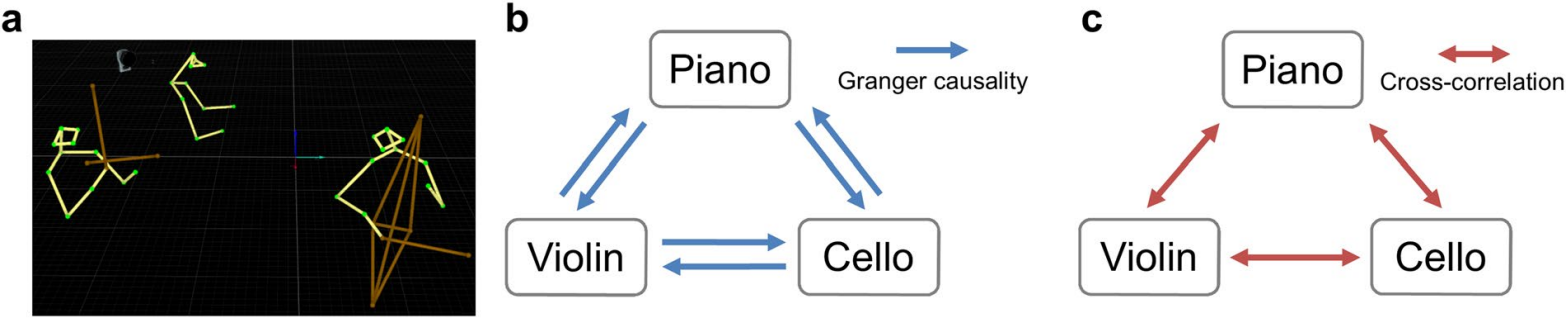
Illustrations of the experimental design and body sway coupling analyses. (**a**) The retroreflective markers were placed on the head and arms of each performer (the green dots connected by the yellow lines) and on the instruments (the brown dots and lines). From the left to the right are the violinist, pianist, and the cellist. The anterior-posterior body sway motion time series for the three performers, based on the markers on their heads, were extracted for subsequent analyses. (**b**) Granger causality of body sway reflects the magnitude of information flow from one performer to another performer. The average of the six unique Granger causalities is the causal density (CD), which represents the average amount of information flow across all possible pairs. (**c**) Cross-correlation of body sway reflects the degree of similarity in each pair of performers. The three unique maximum unsigned cross-correlation coefficients on each trial were averaged for an overall measure of similarity.

For each trial, the recorded motion trajectories were denoised, spatially averaged, down-sampled, z-score–normalized, and projected to the anterior–posterior body orientation to produce three body sway time series, one for each performer (Fig. 1a).

To investigate Granger-coupling of body sway, six unique Granger causalities were obtained from each trial, corresponding to the degree to which the body sway of each performer predicted the body sway of each of the other performers (Fig. 1b). We further averaged these six Granger causalities for each trial as causal density (CD), a composite value representing the average amount of information flow among all possible performer pairs.

**Figure 2:**
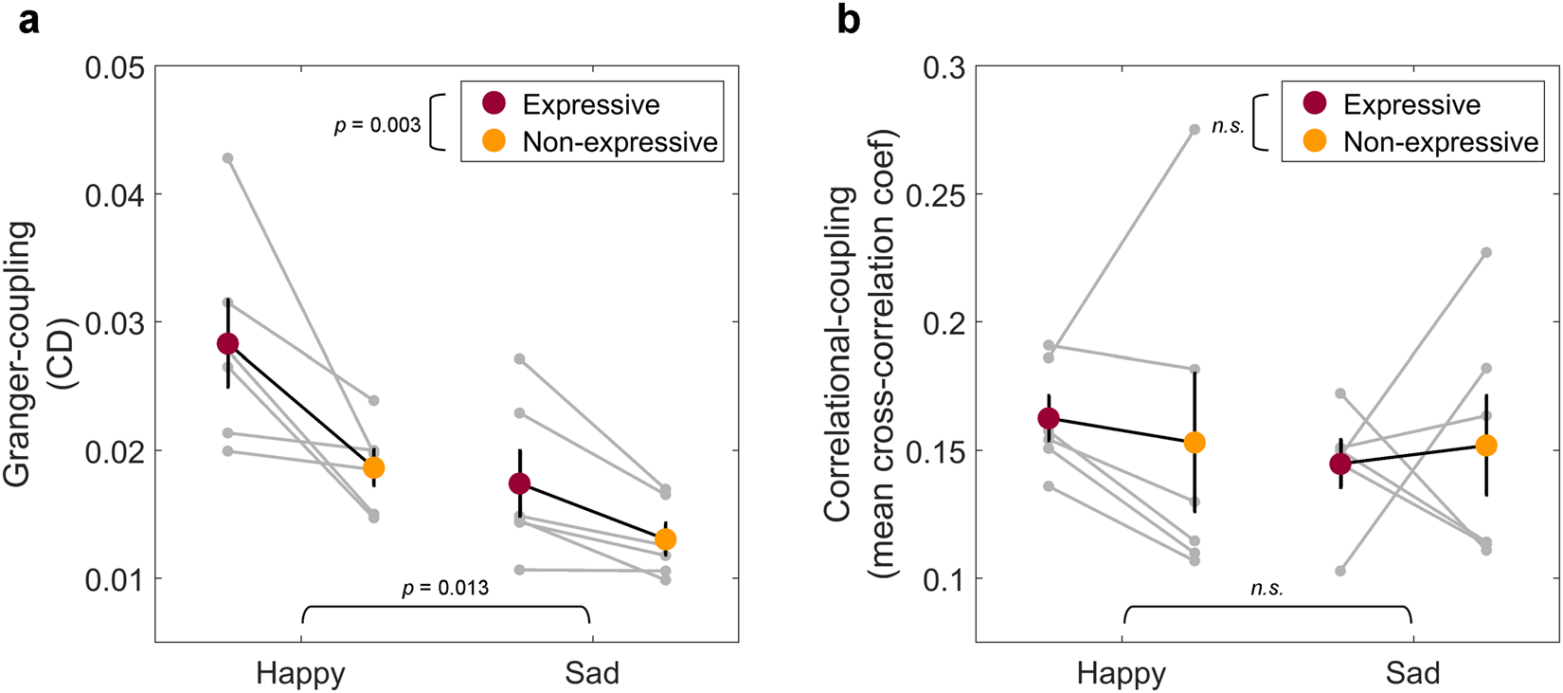
Expressivity and Emotion modulate Granger-coupling of body sway, but not correlational-coupling of body sway. (**a**) A two-way mixed-design ANOVA was conducted on the CD values with Emotion (Happy, Sad) and Expressivity (Expressive, Non-expressive) as factors. The results showed higher CD in the Happy than Sad condition, and higher CD in the Expressive than Non-expressive condition. Each grey dot represents the CD for a trial, and each grey line connects the trials with the same music excerpt under different Expressivity conditions. The red and yellow dots represent the mean CD under Expressive and Non-expressive conditions, respectively. The error bar represents the mean ± one standard error. (**b**) A two-way mixed-design ANOVA was conducted on the mean cross-correlation coefficient with variables as in (**a**). The format is the same as (**a**). The results did not show any significant effect. n.s.: non-significant.

A two-way mixed-design ANOVA was conducted on the CD values with Emotion (*Happy*, *Sad*) and Expressivity (*Expressive*, *Non-expressive*) as factors (Fig. 2a). The results showed a significant main effect of Emotion (*F*(1,10) = 9.23, *p* = 0.013,* η*^2^ = 0.48), with higher CD for *Happy* than *Sad* excerpts. There was also a significant main effect of Expressivity (*F*(1,10) = 14.88, *p* = 0.003,* η*^2^ = 0.60), with higher CD in the *Expressive* than *Non-expressive* conditions. The interaction effect was not significant (*F*(1,10) = 2.13, *p* = 0.175,* η*^2^ = 0.18).

To investigate the correlational-coupling of body sway, we performed the same analyses using cross-correlation, which reflects the similarity between performers’ body sways (Fig. 1c). We took the maximum unsigned cross-correlation coefficient (highest similarity) for each pair of body sways in each trial, and then averaged the coefficients across all pairs within each trial. A two-way mixed-design ANOVA was conducted on the mean cross-correlation coefficients of all body sway pairs (Fig. 2b). There was no significant main effect of Emotion (*F*(1,10) = 0.24, *p* = 0.635,* η*^2^ = 0.02) nor Expressivity (*F*(1,10) = 0.01, *p* = 0.942,* η*^2^ < 0.01), nor was the interaction effect significant (*F*(1,10) = 0.27, *p* = 0.614,* η*^2^ = 0.03).

In sum, the degree of Granger-coupling for body sway among the performers was higher when performers were requested to play the music with emotional expression than without (mechanical, deadpan performance), regardless of whether the pieces were happy or sad. Following the idea that body sway coupling reflects interpersonal information flow^36^, the current findings suggest that jointly expressing emotion in music is associated with more interpersonal information flow among the performers. Furthermore, these effects were only observed in the analyses of Granger-coupling but not correlational-coupling, which is consistent with our previous study^36^.

### Granger-coupling of body sway, but not correlational-coupling, reflects the degree of perceived emotional intensity

**Figure 3:**
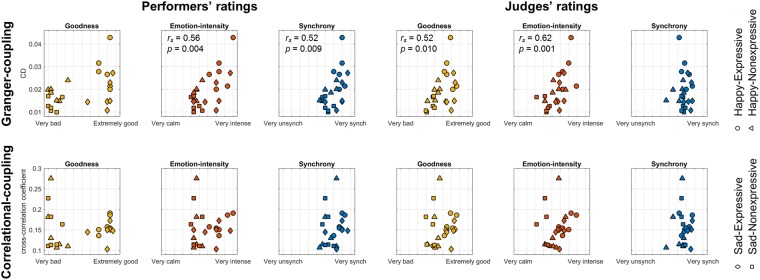
Spearman rank correlation between rated degree of performances and performers’ body sway coupling. The scatter plots in the upper panel represent the Granger-coupling (indexed by CD) of body sway, and plots in the lower panel represent the correlational-coupling (indexed by mean cross-correlation coefficient). Each dot or cross represents the performance of a trial. The dots represent the performers’ self-rated results, and the crosses represent the judges’ rated results. Only the rank correlation coefficients (*r*_*s*_) and the p-values (*p*) of the significant correlations are shown.

To examine how CD and cross-correlation related to qualities of the music produced, we examined correlations with the musicians’ self-ratings of their group performances. Specifically, we first performed Spearman rank correlations between CD of body sway and the average of the performers’ self-ratings for each performance on each of the three measures: how emotionally intense the performance was, how synchronously they played together, and how good the performance was overall (Fig. 3). The results showed that higher CD was significantly correlated with higher self-rated Emotion-intensity (*r*_s_(22) = 0.56, *p* = 0.004) and higher self-rated Synchrony (*r*_s_(22) = 0.52, *p* = 0.009). There was a trend for a correlation between CD and Goodness (*r*_s_(22) = 0.45, *p* = 0.027), but it did not reach the Bonferroni-corrected significance threshold (α = 0.05/3).

To confirm that the associations between CD of body sway and Emotion-intensity and Synchrony levels were not biased by the performers’ subjective experience (such as the fact that they could see each other’s body sway), we recruited additional musicians blind as to the conditions of the study as judges to perform the same ratings based only on the audio recordings. In this case, higher CD was significantly associated with higher Goodness (*r*_s_(22) = 0.52, *p* = 0.010) and Emotion-intensity (*r*_s_(22) = 0.62, *p* = 0.001), but not with Synchrony (*r*_s_(22) = 0.00, *p* = 0.997). This suggests that the degree of body sway coupling among the performers was associated with the degree of emotional intensity being expressed in the music and, further, that this effect was not contingent on observing the participants’ body movements.

We also performed Spearman rank correlation analyses between mean cross-correlation coefficients of all body sway pairs and the performers’ self-ratings (Fig. 3), but the results did not show any significant associations (Goodness: *r*_*s*_(22) = 0.10, *p* = 0.646; Emotion-intensity: *r*_*s*_(22) = 0.21, *p* = 0.336; Synchrony: *r*_*s*_(22) = 0.27, *p* = 0.204). The musician judges’ Goodness (*r*_*s*_(22) = 0.19, *p* = 0.383), Emotion-intensity (*r*_*s*_(22) = 0.28, *p* = 0.178), and Synchrony (*r*_*s*_(22) = −0.03, *p* = 0.900) ratings did not correlate with cross-correlation coefficients, either.

Results showed that CD (Granger-coupling) of body sway was correlated with the degree of emotional intensity as rated by the performers themselves. This correlational effect was replicated in the additional musician judges’ ratings, who were blind to the experimental conditions and only had access to the audio recordings. This suggests that the Granger coupling of body sway was associated with the perceived joint emotional expressivity of the music performances. On the other hand, the CD of body sway was correlated with performer-rated synchrony but not with judge-rated synchrony. As well, CD was correlated with judge-rated goodness but not with performer-rated goodness.

It is important to note that the three correlations are likely to be modulated by additional variables, such as our experimental manipulations of expressivity and emotion (as shown in Fig. 2a), and a more sophisticated approach would perform the correlational analysis within each nested condition. However, we were not able to do so here because the 6 trials within each nested condition were too few. Despite this limitation, at the functional level, the correlational findings suggest an association between CD and perceived level of emotional intensity, and thus the Granger coupling of body sway can be an informative index to reflect joint emotional expression.

### Rated emotion-expression and emotion-valence are consistent with experimental conditions

**Figure 4:**
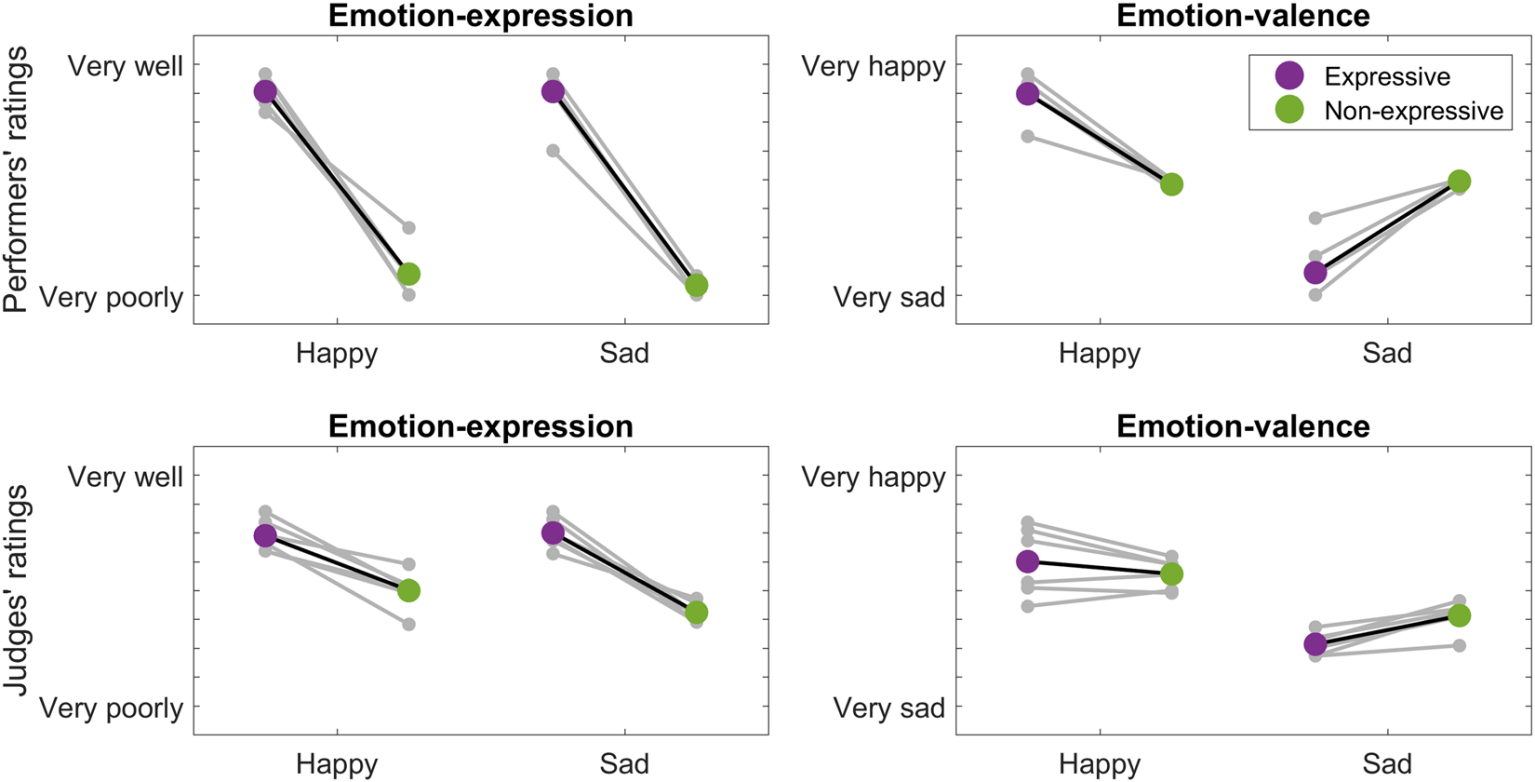
Rated Emotion-expression and Emotion-valence. Two-way mixed-design ANOVAs were conducted on the mean rated Emotion-expression and Emotion-valence with Emotion (Happy, Sad) and Expressivity (Expressive, Non-expressive) as factors. Each grey dot represents the mean rating across raters (performers or external musicians) for each trial, and each grey line connects the trials with the same music excerpt under different Expressivity conditions. The colored dots represent the mean ratings under Expressive and Non-expressive conditions, respectively.

To check whether performers followed the instruction of performing Happy or Sad excerpts with emotional expression or not, we conducted two-way mixed-design ANOVAs on the ratings (Emotion-expression or Emotion-valence) of either the performers themselves or the judges, with Emotion (*Happy*, *Sad*) and Expressivity (*Expressive*, *Non-expressive*) as factors (Fig. 4).

The results of performer-rated Emotion-expression showed a main effect of Expressivity (*F*(1,10) = 407.87, *p* < 0.001,* η*^2^ = 0.98), reflecting that the rated Emotion-expression was higher in the Expressive than the Non-expressive condition. The main effect of Emotion (*F*(1,10) = 0.44, *p* = 0.521,* η*^2^ = 0.04) and the interaction effect (*F*(1,10) = 0.36, *p* = 0.561,* η*^2^ = 0.03) were not significant. These results showed that the rated expressivity was consistent with the experimental condition, confirming that the performers followed the experimental instructions.

The performer-rated Emotion-valence analysis showed a significant interaction effect between Emotion and Expressivity (*F*(1,10) = 128.24, *p* < 0.001, *η*^2^ = 0.93), as well as a significant main effect of Emotion (*F*(1,10) = 131.40, *p* < 0.001, *η*^2^ = 0.93), in which the Happy condition was rated to be more positive (happy) than the Sad condition. The Expression effect was not significant (*F*(1,10) < 0.01, *p* = 0.961,* η*^2^ < 0.01). Furthermore, post-hoc analyses showed that, under the Happy condition, the rated Emotion-valence was more positive (happy) in the Expressive than Non-expressive condition (*t*(5) = 9.09, *p* < 0.001). Conversely, under the Sad condition, the rated Emotion-valence was more negative (sad) in the Expressive than Non-expressive condition (*t*(5) = −7.25, *p* = 0.001). These results confirm that the performances of Happy excerpts expressed greater happiness than performances of Sad excerpts, and this difference was enhanced in the Expressive compared to Non-expressive conditions.

Although the above results showed that the performers’ ratings were consistent with the experimental manipulations, their ratings might have reflected their awareness of the conditions rather than their achievement of these manipulations in their performances. Therefore, we conducted the same analyses on the musician judges’ ratings, who were blind to the experimental conditions.

The results of the musician judges’ ratings of Emotion-expression showed a main effect of Expressivity (*F*(1,10) = 113.14, *p* < 0.001,* η*^2^ = 0.92), whereby their rated Emotion-expression was higher than in the Expressive than Non-expressive conditions. The main effect of Emotion (*F*(1,10) = 2.21, *p* = 0.168,* η*^2^ = 0.18) and the interaction effect (*F*(1,10) = 3.90, *p* = 0.077,* η*^2^ = 0.28) were not significant. These results showed that the expressivity ratings were consistent with the experimental conditions, even when the judges were blind to the experimental conditions.

The musician judges’ ratings of Emotion-valence showed a significant interaction effect between Emotion and Expressivity (*F*(1,10) = 15.80, *p* = 0.003,* η*^2^ = 0.61) and a main effect of Emotion such that excerpts in the Happy condition were rated as more positive than in the Sad condition (*F*(1,10) = 31.05, *p* < 0.001, *η*^2^ = 0.76). The main effect of Expressivity was not significant (*F*(1,10) = 2.50, *p* = 0.145, *η*^2^ = 0.20). Post-hoc paired *t*-tests further showed that the Sad condition was rated more negatively in the Expressive condition than Non-expressive condition (*t*(5) = −5.44, *p* = 0.003), but the same comparison was not significant in the Happy condition (*t*(5) = 1.39, *p* = 0.223). These results confirmed that the performances of Happy excerpts expressed greater happiness than did performances of the Sad pieces. However, in contrast to the performers’ ratings, the rated Emotion-valence was only exaggerated by the Expressivity factor in the Sad condition.

## Discussion

In the present study, we used Granger causality to measure the strength of the predictive relationship between ensemble performers’ body sway while playing with or without emotional expression. Total Granger-coupling of body sway across the ensemble was higher when performing *with expression* than when performing *without expression*. Furthermore, degree of coupling was associated with perceived emotional intensity, both self- and externally-rated. Together, these findings suggest that Granger-coupling of body sway reflects interpersonal joint emotional expression.

Emotionally expressive music performance typically includes larger acoustical variations in features such as tempo, dynamics, and articulation compared to non-expressive performance, in which each performer can mechanically follow their music score^18^. Coordinating these expressive nuances presumably requires greater communication amongst performers than deadpan performance. It is possible that Granger-coupling of body sway reflects the nonverbal interpersonal coordination required to achieve complex cohesive joint emotional expression in ensemble music performances.

In the analysis, Granger-coupling of body sway reflected both the degree of emotional expression and the intended emotion, such that Granger-coupling tended to be higher when performing *happy* excerpts than *sad* excerpts. We did not have a specific hypothesis about this outcome, but it appears that performing happy pieces may require a higher level of coordination (and therefore information flow) among performers. Happy and sad pieces are known to be qualitatively different in many compositional and expressive aspects^16^. For example, pieces perceived as *happy* tend to be faster and louder than pieces perceived as *sad*^52^, and these factors might require a higher degree of coordination, which in turn might affect the level of information flow between performers. Future studies are needed to investigate the relationship between performance factors, acoustic features, emotional valence, and music ensemble coordination.

The association between Granger-coupling of body sway and the perceived emotional intensity ratings suggests that body sway reflects the performance of joint emotional expression. Importantly, this association was replicated by musician judges’ ratings on the audio recordings without access to the video recordings, which suggests that the perceived emotional intensity was not confounded with visual information about the body sway or knowledge of the intentions of the performers. This evidence directly supports the argument that Granger-coupling of body sway reflects joint emotional expression.

The performers’ and judges’ ratings on synchrony and goodness showed different correlational patterns with Granger-coupling of body sway, suggesting that performers and listeners may perceive the interactions between musicians somewhat differently. It should also be noted that performers could see each other whereas listeners made their ratings on the basis of audio files alone. It remains for future research to investigate both performer/audience differences and audiovisual/audio-alone differences more fully.

The current findings provide further evidence that Granger-coupling of body sway among co-actors reflects higher-order aspects of joint action. Body sway is a global measurement reflecting the summation of all actions of an individual^29^, and it does not precisely time-lock to the individual and local actions required to produce a joint action. Coupling of body sway among co-actors has been shown to reflect performance across many joint action tasks, including motor coordination tasks^26,27^, conversation^28–30^, and music ensemble performance^25,31–33^. Consistent with this idea, our previous study showed that the degree of predictive information flow (Granger-coupling) of body sway, but not necessarily the degree of temporal synchrony (correlational-coupling) of body sway, reflects the perceived quality of performance^36^. Findings from the present study suggest that emotional expression may underlie the link between body sway and performance quality observed in the previous work.

We additionally performed cross-correlation analyses on body sway (correlation-coupling). We found no evidence that performing with or without expression modulates correlational-coupling, nor did the correlational-coupling associate with any of the performance ratings. Granger causality and cross-correlation are distinct time series analyses. Granger causality measures the strength of one variable predicting another variable, over and above the degree to which it is predicted by its own prior time series, and it is often interpreted as an index of information flow. On the other hand, cross-correlation measures the similarity of two time series shifted in time with respect to each other. It is important to note that cross-correlation does not reflect information flow because it is essentially a similarity measurement^44^. This comparison is consistent with our previous finding that Granger-coupling of body sway of string quartet performance reveals leader-follower information flow for coordination, but correlational-coupling does not^36^. While it has been reported that body sway similarity in piano duets is associated with event-to-event acoustic synchrony^47^, evidence from our current and previous studies suggests that body sway among the performers also reflects information flow. Moreover, the magnitude of information flow is associated with higher-order aspects of interpersonal coordination, such as leader-follower relationships^36^ and joint emotional expression, rather than sensorimotor event-to-event temporal synchronization, such as piano keystrokes.

The present study provides a novel basis for investigating emotional expression as a joint action goal. It is important to note that the expression of an emotion does not necessarily assume that the emotion is felt by the co-actor. However, previous work has shown that emotional factors, such as the emotional status of each co-actor, influence sensorimotor joint coordination performance^6,11^. It is not yet clear whether co-actors’ personal emotional status affects joint emotional expression, and further study is needed to investigate the relationship between felt emotion and joint emotion expression.

To our knowledge, the present study is the first to explicitly examine joint emotional expression across co-actors. We show that it is possible to measure the degree of emotional expression coordinated among the ensemble members by quantifying the degree of information flow between individuals’ body sways. Although the current study was limited to a music ensemble as a model, we speculate that the current findings are generalizable to other forms of joint action, given that music performance and other forms of joint action tasks share many psychological principles^42^. Future work is needed, however, to examine the extent to which predictive movement between and among co-actors characterizes the quality of joint actions in broader contexts – for example, creativity of interpersonal collaboration^53^, mother-infant dyads^54^, or even applications for social intervention for children with Autism spectrum disorder^55^. Overall, we show that body sway coupling is associated with joint emotional expression in a music ensemble, and provide a new way to examine joint expression across co-actors more generally.

## Methods

### Participants

The participants were members of the Gryphon Trio, an internationally acclaimed Canadian professional music ensemble, which includes one pianist (M, age = 53 years), one violinist (F, age = 49 years), and one cellist (M, age = 50 years).

Eleven additional internationally acclaimed professional musicians (two pianists, four violinists, two violists, and three cellists; three men and eight woman; mean age = 43.4 years, range = 34–58 years) were recruited as judges.

All trio performers and musician judges had normal hearing and were neurologically healthy by self-report. Informed consent was obtained from each participant, and they received reimbursement. All procedures were approved by the McMaster University Research Ethics Board, and all methods were performed in accordance with the approved guidelines and regulations.

### Stimuli and Apparatus

The data were collected in the McMaster University Large Interactive Virtual Environment Laboratory (LIVELab; livelab.mcmaster.ca). The trio performed six happy and six sad excerpts (Table 1). The authors and trio performers chose the excerpts together from the trio’s current repertoire based on the criteria that the excerpts had (1) high emotional expressivity, (2) clear happy or sad emotion, and (3) balanced roles among music parts (i.e., each part was approximately equally prominent, rather than a prominent distinction between the melody and accompanying parts). We selected pieces from Classical (Beethoven), Romantic (Dvořák), and Tango (Piazzolla) styles so our findings could be generalized to a broad range of Western music styles. In the Happy condition, performers only played pieces that were determined a priori by the performers and experimenters as communicating happiness; likewise, in the Sad condition, pieces were determined a priori as communicating sadness. We did not control the acoustic characteristics (e.g., tempo, number of notes) between the happy and sad excerpts, as we aimed to keep the performances as naturalistic as possible. However, it should be noted that the same pieces were played in the expressive and non-expressive conditions, so this would not affect the main comparison between these conditions.

**Table 1:** Trial order and experimental conditions.

Trial	Emotion	Expressivity	Piece	Measure numbers
1	Happy	Expressive	Dvořák: Dumky Trio, mvt 1	35–72
2	Happy	Non-expressive	Dvořák: Dumky Trio, mvt 1	35–72
3	Sad	Non-expressive	Dvořák: Dumky Trio, mvt 1	11–34
4	Sad	Expressive	Dvořák: Dumky Trio, mvt 1	11–34
5	Happy	Non-expressive	Dvořák: Dumky Trio, mvt 3	43–69
6	Happy	Expressive	Dvořák: Dumky Trio, mvt 3	43–69
7	Sad	Expressive	Dvořák: Dumky Trio, mvt 2	1–41
8	Sad	Non-expressive	Dvořák: Dumky Trio, mvt 2	1–41
9	Happy	Expressive	Dvořák: Dumky Trio, mvt 4	89–122
10	Happy	Non-expressive	Dvořák: Dumky Trio, mvt 4	89–122
11	Sad	Non-expressive	Dvořák: Dumky Trio, mvt 4	1–22
12	Sad	Expressive	Dvořák: Dumky Trio, mvt 4	1–22
13	Happy	Non-expressive	Dvořák: Dumky Trio, mvt 6	132–206
14	Happy	Expressive	Dvořák: Dumky Trio, mvt 6	132–206
15	Sad	Expressive	Piazzolla: Oblivion	Entire piece
16	Sad	Non-expressive	Piazzolla: Oblivion	Entire piece
17	Happy	Expressive	Piazzolla: Otoño Porteño	78–102
18	Happy	Non-expressive	Piazzolla: Otoño Porteño	78–102
19	Sad	Non-expressive	Piazzolla: Primavera Porteña	59–89
20	Sad	Expressive	Piazzolla: Primavera Porteña	59–89
21	Happy	Non-expressive	Beethoven: Op. 97 Scherzo	1–125
22	Happy	Expressive	Beethoven: Op. 97 Scherzo	1–125
23	Sad	Expressive	Piazzolla: Milonga del Ángel	Entire piece
24	Sad	Non-expressive	Piazzolla: Milonga del Ángel	Entire piece

A passive optical motion capture system (24 Oqus 5 + cameras and an Oqus 210c video camera; Qualisys) recorded the head movements of participants at 120 Hz. Each participant wore a cap with four retroreflective markers (3 mm) placed on the frontal-midline, centre-midline, and above the left and right ears. Three positional markers were placed on the ground to calibrate the anterior-posterior and left-right axes of each performer’s body. Additional markers placed on the arms and instruments were not analyzed in the current study. The performers confirmed that these placements did not constrain their body movements and that they were able to perform as usual.

The music performances were audio recorded using two DPA 4098-DL-G-B01–015 microphones suspended above the trio, digitized at 48 kHz/24 bit using Reaper recording software (Cockos, Inc.).

### Design and Procedure

A factorial design was used, with Emotion (*Happy*, *Sad*) and Expressivity (*Expressive*, *Non-expressive*) as factors. In the *Expressive* condition, performers were requested to play the excerpts emotionally expressively, as they would in a typical music performance. In contrast, in the *Non-expressive* condition, performers were requested to play the excerpts without emotional expression (deadpan or mechanical performance). In both conditions, performers were asked to play the excerpts as best as they could under the given condition, and the performers were aware that their performances would be recorded and rated. Within each trial, an excerpt was played for a total of three minutes. To make every trial three minutes long, if the performance of an excerpt was shorter than three minutes, the performers looped their performance from the beginning until the three-minute mark was reached. This was necessary to collect enough data points for the time series analyses.

The complete design is shown in Table 1. Each excerpt was performed twice in consecutive trials, once in the *Expressive* condition and once in the *Non*-*expressive* condition. All the conditions were counterbalanced. There were no practice trials, but the performers were already familiar with the pieces. The entire experiment, including preparation, took approximately four hours and was completed on the same day.

Once a three-minute trial ended, each performer independently rated five aspects of the group’s performance using a 9-point Likert scale (Low: 1 to High: 9). (1) Goodness (“How good was it in general?”), (2) Emotion-expression (“How well was the emotion expressed?”), (3) Emotion-valence (“How sad-happy was the emotion expressed?”), (4) Emotion-intensity (“How intense-calm was the emotion expressed?”), and (5) Synchrony (“How technically synchronized was it?”). Because the ensemble was comprised of high-level, professional musicians who had performed together for many years, we expected that they would be sensitive judges of these variables.

Additional professional musician judges independently rated each of the trio’s performances using the same questionnaire. These judges conducted their ratings solely based on the audio recordings at home at their convenience. The purpose of the study and the identities of the trio performers were not revealed to the raters.

### Motion capture data processing

The motion capture data processing was similar to our previous study^36^. Motion trajectories were exported from Qualisys Track Manager for processing and analysis in MATLAB. The first 180 s of each excerpt were analyzed. Missing data due to recording noise were found in only 15 of 864 trajectories and for durations shorter than 6 ms. These durations were gap filled with spline interpolation. Each trajectory was down-sampled to 8 Hz by spatially averaging the samples within each nonoverlapped 125-ms window. This was done because Granger causality analysis prefers a low model order for capturing a given physical time length of the movement trajectory^56^. Visual inspection confirmed that this rate was sufficient for capturing most head movements. No filtering or temporal smoothing was applied to the data because temporal convolution distorts the estimation of Granger causality^56^. To estimate the anterior–posterior body sway, we spatially averaged the positions of the four motion capture markers on the head of each performer in the x–y plane (collapsing altitude) for each time frame, and the anterior–posterior orientation was referenced to the surrounding markers placed on the ground. Finally, each time series was normalized (z-scaled) to equalize the magnitude of the sway motion among performers. This procedure produced three normalized body sway time series, one for each performer for each trial.

### Granger causality of body sway

The MATLAB Multivariate Granger Causality (MVGC) Toolbox^56^ was used to estimate the magnitude of Granger causality between each pair of body sway time series among all three performers in each quartet. First, the MVGC toolbox confirmed that each time series passed the stationary assumption for Granger causality analysis, with the spectral radius less than 1. Second, the optimal model order (the length of history included) was determined by the Akaike information criterion on each trial. The optimal model order is a balance between maximizing goodness of fit and minimizing the number of coefficients (length of the time series) being estimated. The model order used was 14 (1.75 s) because this was the largest optimal model order across trials within the trio. Model order was fixed (i.e., did not vary by trial), which avoided model order affecting Granger causalities differently on different trials, and the largest model order across trials covered all optimal model orders across trials. In this way, six unique Granger causalities were obtained for each trial, corresponding to the degree to which each of pianist, violinist, and cellist predicted each of the other two performers. It is important to note that we estimated each Granger causality between two time series conditional on the remaining one time series because, in this way, any potential common influence on other variables was partialed out^56^. We further averaged these six unique Granger causalities for each trial as causal density (CD), which represents the total amount of information flow within the ensemble^57^. We did not analyze each Granger causality separately because we were interested in how the total directional information flow within the ensemble was influenced by the independent variables Emotion and Expressivity.

### Cross-correlation of body sway

Cross-correlation quantifies the similarity between two time series as a function of a shifting time step. To empirically compare Granger causality and cross-correlation, we performed cross-correlation analyses on the same preprocessed data to which we had applied Granger causality, and the cross-correlation coefficients were calculated for the window up to plus or minus the model order that was used for the Granger causality. Although the window size was optimized for Granger causality, it would not suboptimize the cross-correlation analyses, as the window size (1.75 s) was actually wider than that used in most of the cross-correlation analyses on music performers’ body sway^25,47^, which has typically ranged up to ± one beat. Within the window we picked, the maximum unsigned cross-correlation coefficient (highest similarity) for each of the three pairs of musicians for each trial, and then averaged the coefficients across all pairs within each trial.

### Statistical analyses

We performed mixed-design ANOVAs separately on CD and cross-correlation coefficients values to analyze the modulation of body sway coupling by Emotion (*Happy*, *Sad*) and Expressivity (*Expressive* and *Non-expressive*). The significance of the effects was determined with type-II Wald tests using the “Anova” function in the “car” package in R^58^.

We considered Emotion of the music excerpts (*Happy*, *Sad*) as a random-effect and Expressivity as a fixed-effect. Traditional approaches would treat Emotion as a fixed-effect. However, as happy and sad are characteristics of the stimuli, and we are using a small sample of all possible happy and sad stimuli, ignoring the sampling variance of these few samples could potentially affect the generalizability of the reported effect to the entire population of happy and sad stimuli. Therefore, it has been proposed that it is better to treat stimulus characteristics as random effects^59,60^.

To investigate whether CD and cross-correlation coefficients reflected expressive aspects of the performances, we performed Spearman rank correlation analyses between the CD and cross-correlation coefficients separately with the subjective ratings of the performances both by the trio performers and by the judges.

Every statistical test was performed two-tailed. We set α = 0.05, and Bonferroni-adjusted α was used for each post hoc comparison series as a conservative control for type I error.

## Data Availability

The datasets generated and/or analyzed during the current study are available from the corresponding author on reasonable request.
